# Patient-Reported Trajectories After Intestinal Stoma Formation: A Prospective Longitudinal Study of Stoma-Related Outcomes and Global Quality of Life

**DOI:** 10.3390/jcm15218654

**Published:** 2026-11-06

**Authors:** Filippo Carannante, Asia Rizzini, Valentina Miacci, Francesca Carnevale, Paula Elena Papuc, Fausto D’Agostino, Gianluca Costa, Gabriella Teresa Capolupo

**Affiliations:** 1 UOC Chirurgia Colorettale, Fondazione Policlinico Universitario Campus Bio-Medico, Via Àlvaro del Portillo 200, 00128 Rome, Italyvalentina.miacci@policlinicocampus.it (V.M.);; 2 Research Unit of Surgery, Department of Medicine and Surgery, Università Campus Bio-Medico di Roma, Via Alvaro del Portillo 21, 00128 Rome, Italy; 3 UOC Anestesia e Rianimazione, Fondazione Policlinico Universitario Campus Bio-Medico, Via Álvaro del Portillo 200, 00128 Rome, Italy; 4 Department of Life Science, Health and Health Professions, LINK Campus University, 00165 Rome, Italy

**Keywords:** intestinal stoma, ileostomy, colostomy, patient-reported outcomes, quality of life, longitudinal study, postoperative recovery

## Abstract

**Background**: Recovery after intestinal stoma formation involves practical, psychological, social, and functional changes that may not evolve in parallel with patients’ overall perception of quality of life. This study examined patient-reported trajectories during the first six postoperative months and explored whether multidimensional stoma-related outcomes followed the same pattern as directly reported global quality of life. **Methods**: This prospective single-centre observational study included patients with complete assessments at approximately 2 weeks, 1 month, 3 months, and 6 months after ileostomy or colostomy formation. Patient responses were collected at each follow-up and subsequently manually transcribed into an electronic study database. The primary exploratory outcome was a corrected 30-component stoma-related patient-reported composite index (range 0–30; higher values more favourable) derived from an adapted, non-validated questionnaire. A direct 0–10 global quality-of-life item, which also contributed one component to the composite, was analysed separately. Longitudinal change was assessed using Friedman tests with Bonferroni-adjusted Wilcoxon signed-rank comparisons. Post hoc robustness analyses recomputed the composite after excluding the global quality-of-life component and after additionally excluding the sexual-activity components. **Results**: Our study included 23 patients. The median composite index increased from 9.4 (IQR 7.2–13.6) at 2 weeks to 10.9 (8.7–13.6) at 1 month and 12.2 (9.3–15.3) at 3 months and was 11.8 (9.3–17.2) at 6 months (Friedman chi-square(3) = 19.08, *p* < 0.001; Kendall’s W = 0.276). Bonferroni-adjusted comparisons were significant for 2 weeks versus 3 months (*p* = 0.037), 2 weeks versus 6 months (*p* = 0.016), and 1 month versus 6 months (*p* = 0.045). Median global quality-of-life ratings were 5, 6, 6, and 5, respectively, with no significant longitudinal change (*p* = 0.753). Excluding the global quality-of-life component from the composite produced a similar overall result (Friedman chi-square(3) = 19.38, *p* < 0.001). **Conclusions**: The exploratory composite showed statistically significant longitudinal change during the first six postoperative months, whereas directly reported global quality of life did not show a parallel change. Because the composite is non-validated and no threshold for clinically meaningful change has been established, these findings should not be interpreted as demonstrating clinically meaningful improvement in adaptation or quality of life. Rather, they suggest that several stoma-related patient-reported dimensions may change over time differently from global quality of life. These findings are exploratory and require confirmation in larger cohorts using validated patient-reported outcome measures.

## 1. Introduction

Intestinal stoma formation can profoundly affect physical functioning, body image, social participation, intimate relationships, daily activities, and psychological well-being. In patients with colorectal cancer, recent systematic evidence indicates an overall adverse association between ostomy formation and quality of life, with psychosocial and emotional difficulties contributing substantially to this burden [1].

The impact of a stoma is inherently multidimensional. The City of Hope Quality of Life-Ostomy Questionnaire [2] was developed to assess physical, psychological, social, and spiritual dimensions of quality of life in individuals living with an ostomy and demonstrated satisfactory reliability and construct validity in its original psychometric evaluation. Other stoma-specific instruments have subsequently been developed and validated. In an Italian multicentre population, Canova et al. [3] confirmed the validity of a 20-item stoma-specific quality-of-life instrument in patients with colostomies and ileostomies. These findings highlight the importance of patient-reported outcomes that capture stoma-specific experiences rather than relying exclusively on a single global measure of health status.

Recovery after a newly formed stoma is dynamic and may not follow the same trajectory across individual domains or in parallel with overall health-related quality of life. Prospective evidence remains comparatively limited. Stott et al. [4] demonstrated that several dimensions of adjustment may persist for months following stoma formation, with improvement occurring at different rates across individual domains. Similarly, a longitudinal study in colorectal cancer patients showed pronounced impairment in global health and functional outcomes during the early postoperative period, with some deficits persisting at subsequent assessments [5]. Qualitative evidence further suggests that recovery involves the interaction of practical self-management, changes in body image, social participation, uncertainty, and psychological adaptation rather than a single uniform process [6].

Understanding this trajectory is clinically relevant because acquisition of technical competence in stoma management may not necessarily coincide with broader functional or psychosocial recovery. The present prospective longitudinal study therefore aimed to describe patient-reported changes during the first six months after intestinal stoma formation. The primary objective was to assess longitudinal change in an exploratory multidimensional stoma-related patient-reported composite index reconstructed from the study questionnaire. A direct 0–10 rating of overall quality of life was analysed separately to examine whether stoma-related patient-reported changes were accompanied by a parallel change in global quality of life. Secondary exploratory analyses examined sex and age, while stoma type, surgical setting, selected functional and psychosocial outcomes, and self-reported stoma-related complications were described.

## 2. Methods

### 2.1. Study Design and Participants

This was a prospective, single-centre observational study conducted at the Colorectal Surgery Unit of Policlinico Campus Bio-Medico, Rome, Italy. Adult patients undergoing surgical creation of an intestinal stoma were prospectively enrolled between January 2024 and December 2025.

Patients were eligible if they were aged 18 years or older, underwent creation of an ileostomy or colostomy in either an elective or emergency setting, and were able to understand and complete the study questionnaire in Italian. Both temporary and permanent stomas were eligible, irrespective of the underlying surgical indication. Although both temporary and permanent stomas were eligible according to the study protocol, all patients included in the present complete-case longitudinal analysis had a temporary stoma. All four questionnaire assessments included in the analysis were completed while the stoma remained in situ; no post-reversal questionnaire was included in the longitudinal dataset. In our clinical practice, temporary stomas are generally considered for reversal from approximately 12 weeks after formation when clinically appropriate; however, this is not a fixed deadline, and the actual timing varies according to the underlying disease, postoperative course, ongoing treatment, patient condition, and other clinical factors.

All patients included in the longitudinal analysis had their intestinal stoma in place at each scheduled assessment, including the 3-month follow-up. No patient-reported outcomes collected after stoma reversal were included in the analysis. Although the stomas were intended to be temporary, the timing of reversal was determined individually according to postoperative recovery, underlying disease, adjuvant treatment requirements, and clinical eligibility. Therefore, the 3-month assessment specifically reflects the experience of patients who were still living with a stoma at that time.

Patients were excluded in the presence of cognitive or neurological impairment interfering with questionnaire completion, severe psychiatric disorders compromising reliable participation, inability to provide informed consent, or refusal to participate.

The present manuscript analysis was restricted to the patients with complete questionnaire data at all four postoperative assessments, corresponding to 92 observations. The analytical file available for reanalysis did not permit reliable reconstruction of the number of patients originally approached or enrolled but not represented in the complete-case dataset, or the reasons for incomplete follow-up. Accordingly, the study should be interpreted as a complete-case exploratory analysis, and the potential for selection and attrition bias should be acknowledged. Given the exploratory nature of the study and its single-centre design, no formal a priori sample-size calculation was performed.

### 2.2. Questionnaire and Data Collection

Patient-reported outcomes were collected using a structured 59-item questionnaire developed from the City of Hope Quality of Life-Ostomy Questionnaire framework [2] and adapted to the Italian clinical context. The questionnaire included demographic and clinical characteristics, lifestyle changes, sexual activity, dietary and clothing adaptations, practical aspects of stoma management, social and psychological functioning, and self-reported stoma-related complications. Because the adapted questionnaire was not formally validated in this form, its individual items and the derived composite were treated as exploratory patient-reported measures rather than as a validated quality-of-life instrument.

Patient-reported assessments were performed prospectively at four predefined postoperative intervals: approximately 2 weeks after stoma creation and at 1, 3, and 6 months. Responses from the individual follow-up assessments were subsequently manually transcribed into a structured electronic study database. Electronic database timestamps therefore reflect the date of data entry and were not used to assign follow-up timing. Longitudinal timepoints were assigned according to the postoperative assessment to which each response belonged.

### 2.3. Primary Outcome: Exploratory Stoma-Related Patient-Reported Composite Index

The primary outcome for the present manuscript was an exploratory stoma-related patient-reported composite index reconstructed from the scoring logic implemented in the original study spreadsheet. The component structure, direction of scoring, and equal weighting of components were inherited from this pre-existing scoring framework rather than selected according to the longitudinal results observed in the present reanalysis. Reconstruction consisted of mapping the spreadsheet scoring rules to the corresponding item-level questionnaire responses and auditing these rules against the wording and response categories of the original questionnaire. No data-driven reweighting or optimisation of components according to the observed longitudinal results was performed. Thirty-one questionnaire items contributed to 30 scoring components because the two items assessing sexual activity before and after stoma creation were combined into a single conditional component.

Each component was rescaled from 0 to 1, where 0 represented a less favourable and 1 a more favourable response according to the original scoring direction. Dichotomous responses were coded as 0 or 1. Numerical 0–10 items were divided by 10 when higher raw scores represented more favourable responses or transformed as 1 − raw score/10 when higher raw scores represented greater symptoms or limitation. Ordinal items were assigned intermediate values where specified by the scoring algorithm. Components were equally weighted and summed to obtain a total index ranging from 0 to 30. The complete item-level scoring rules are provided in Supplementary Table S3. The sexual-satisfaction component classified an affirmative response as favourable and other/non-applicable responses as 0, according to the original spreadsheet scoring logic. Non-applicability does not necessarily represent an unfavourable patient-reported outcome. To avoid additional analytical discretion from retrospectively redefining this component, the original reconstructed coding was retained for the primary analysis. Its potential influence was explored in a post hoc robustness analysis excluding both sexual-activity components together with the global QoL component.

During the audit of the reconstructed scoring algorithm, one spreadsheet string-matching error was identified in the item assessing psychological limitation in pursuing hobbies or sports. Affirmative responses contained additional text and had therefore been incorrectly classified as favourable by the original exact-match formula. This was treated as a coding error rather than a modification of the scoring construct based on the longitudinal results, and all affirmative responses were consistently recoded as indicating limitation. To evaluate whether this correction influenced the primary longitudinal finding, a post hoc robustness analysis also excluded this component entirely.

Because the questionnaire had been adapted from an established instrument but the modified version and the composite index had not undergone formal psychometric validation, the index was treated exclusively as an exploratory multidimensional patient-reported measure. It should not be interpreted as a validated quality-of-life, adjustment, or adaptation scale. The equal weighting of heterogeneous components was inherited from the pre-existing spreadsheet scoring framework and was not based on psychometric validation or optimisation against the observed outcomes.

### 2.4. Secondary and Exploratory Outcomes

A direct questionnaire item asking patients to rate their overall quality of life on a 0–10 scale was analysed separately as a key secondary outcome. This global QoL item also contributed one of the 30 components to the composite index and was therefore not statistically independent of the primary outcome. To assess whether the longitudinal composite finding was driven by this overlap, a post hoc robustness analysis recomputed the composite after excluding the global QoL component.

Exploratory analyses examined the relationship between the composite index and sex and age. Stoma type and elective versus emergency surgery were described as exploratory clinical subgroups. Selected questionnaire items were reported descriptively to characterise practical and psychosocial experiences, including ability to manage the stoma outside the home, limitation of leisure activities, psychological and physical limitation of hobbies or sports, and interest in peer-support groups.

Early and late stoma-related complications were described. When the same complication was reported at more than one follow-up assessment, it was counted once at the patient level for the overall complication analysis.

### 2.5. Data Management

All analyses were reconstructed from the original longitudinal patient-level dataset. Each patient was assigned a unique study identifier, and the four repeated observations were linked at the individual level. Time-invariant demographic and clinical variables were defined using baseline records. Derived worksheets previously created for subgroup analyses were not used for the final analysis.

The analytical dataset was locked after data cleaning, verification of longitudinal patient matching, reconstruction of the composite scoring algorithm, and correction of response coding. Because patient responses had been manually transcribed into the electronic database, transcription error was considered a potential source of measurement error; the manuscript reanalysis therefore used an audited patient-level dataset rather than previously derived subgroup worksheets.

### 2.6. Statistical Analysis

The primary analysis evaluated longitudinal change in the corrected composite index across the four postoperative timepoints using the Friedman test for repeated measures. Following a statistically significant overall test, six pairwise comparisons were evaluated using Wilcoxon signed-rank tests: 2 weeks versus 1 month, 2 weeks versus 3 months, 2 weeks versus 6 months, 1 month versus 3 months, 1 month versus 6 months, and 3 months versus 6 months. Bonferroni correction was applied across the six comparisons. The magnitude of the overall longitudinal effect was summarized using Kendall’s coefficient of concordance (W).

Because of the limited sample size and the distributional characteristics of the composite outcome, continuous variables were primarily summarised using the median and interquartile range (IQR). Minimum and maximum values were reported where informative. Categorical variables were summarised as frequencies and percentages.

The direct 0–10 overall QoL item was analysed longitudinally using a separate Friedman test. Post hoc robustness analyses repeated the primary longitudinal analysis after (1) excluding the global QoL component from the composite, (2) excluding the global QoL component together with the two sexual-activity components, and (3) excluding the corrected psychological hobby/sport limitation component. For the exploratory sex analysis, one longitudinal summary value was calculated for each patient by averaging composite-index values across the four assessments. Male and female patients were compared using the Mann–Whitney U test, with rank-biserial correlation as an effect-size measure; a sensitivity analysis repeated this comparison after excluding the sexual-activity components. Age was analysed continuously using Spearman’s rank correlation coefficient.

Analyses according to stoma type and surgical setting were considered descriptive because of the limited subgroup sizes. Individual questionnaire items and stoma-related complications were likewise analysed descriptively, without item-by-item inferential testing, to avoid excessive multiple comparisons.

All statistical tests were two-sided. A *p*-value < 0.05 was considered statistically significant for the primary overall analysis. Interpretation of the six pairwise longitudinal comparisons was based on Bonferroni-adjusted p-values. Secondary, subgroup, and robustness analyses were exploratory and were interpreted with emphasis on effect size, consistency, and clinical plausibility. The locked reanalysis was performed in Python 3.13.5 using SciPy 1.17.0 and NumPy 2.3.5.

## 3. Results

### 3.1. Study Population

The analytic cohort comprised 23 patients who completed all four postoperative assessments, corresponding to 92 longitudinal observations. Thirteen patients (56.5%) were female and 10 (43.5%) were male. Median age at stoma formation was 63 years (IQR 52–71; range 26–87), and median body mass index was 23.4 (IQR 20.2–25.6).

Sixteen patients (69.6%) had an ileostomy and seven (30.4%) a colostomy. Surgery was elective in 16 patients (69.6%) and performed in an emergency setting in seven (30.4%). After harmonisation of the free-text diagnostic fields, the indication for stoma formation was oncological disease in 16 patients (69.6%), inflammatory bowel disease in four (17.4%), and diverticular disease in three (13.0%) (Table 1). All 23 patients (100%) had temporary stomas, and all four assessments were completed with the stoma in situ.

### 3.2. Longitudinal Change in the Exploratory Composite Index

The corrected exploratory stoma-related patient-reported composite index differed significantly across the four postoperative assessments (Friedman chi-square(3) = 19.08, *p* < 0.001), with Kendall’s W = 0.276.

**Figure 1:**
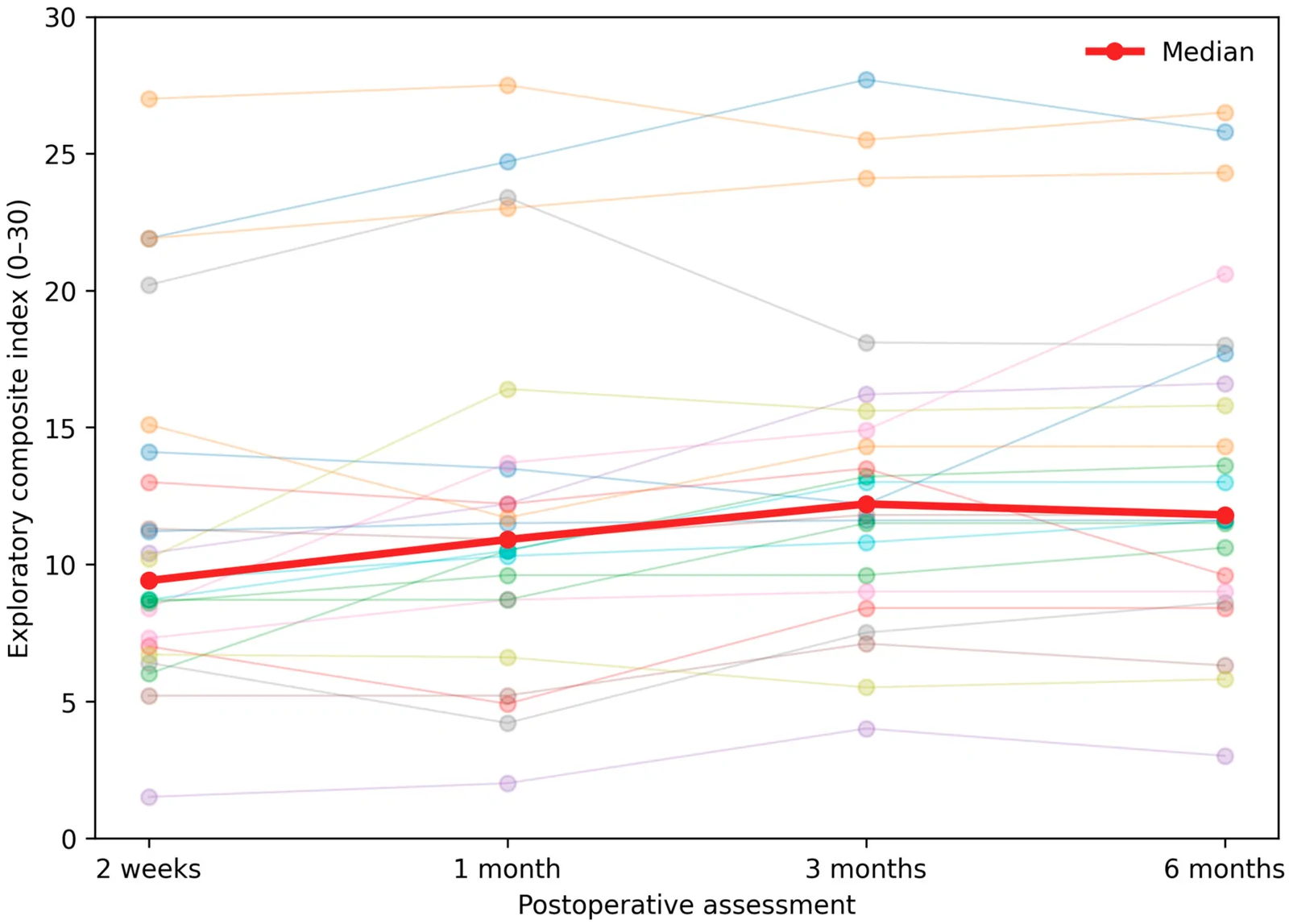
Individual longitudinal trajectories of the corrected exploratory stoma-related patient-reported composite index among 23 patients with temporary intestinal stomas. Assessments were performed at 2 weeks, 1 month, 3 months, and 6 months after stoma formation, with the stoma in situ at all time points. Thin lines represent individual patient trajectories, while the bold line represents the cohort median. Higher index values indicate more favourable patient-reported responses according to the scoring algorithm. The composite index is exploratory and has not undergone formal psychometric validation.

The median index value was 9.4 (IQR 7.2–13.6) at 2 weeks, 10.9 (IQR 8.7–13.6) at 1 month, 12.2 (IQR 9.3–15.3) at 3 months, and 11.8 (IQR 9.3–17.2) at 6 months (Table 2; Figure 1).

Bonferroni-adjusted post-hoc comparisons showed significantly higher scores at 3 months compared with 2 weeks (adjusted *p* = 0.037) and at 6 months compared with 2 weeks (adjusted *p* = 0.016). Scores at 6 months were also significantly higher than at 1 month (adjusted *p* = 0.045). The remaining pairwise comparisons did not reach statistical significance after correction for multiple testing, including 2 weeks versus 1 month (adjusted *p* = 0.441), 1 month versus 3 months (adjusted *p* = 0.111), and 3 months versus 6 months (adjusted *p* = 1.000).

Individual trajectories were heterogeneous, with substantial between-patient variability at all assessment points and greater dispersion at the later follow-up assessments (Figure 1).

### 3.3. Global Quality of Life

**Figure 2:**
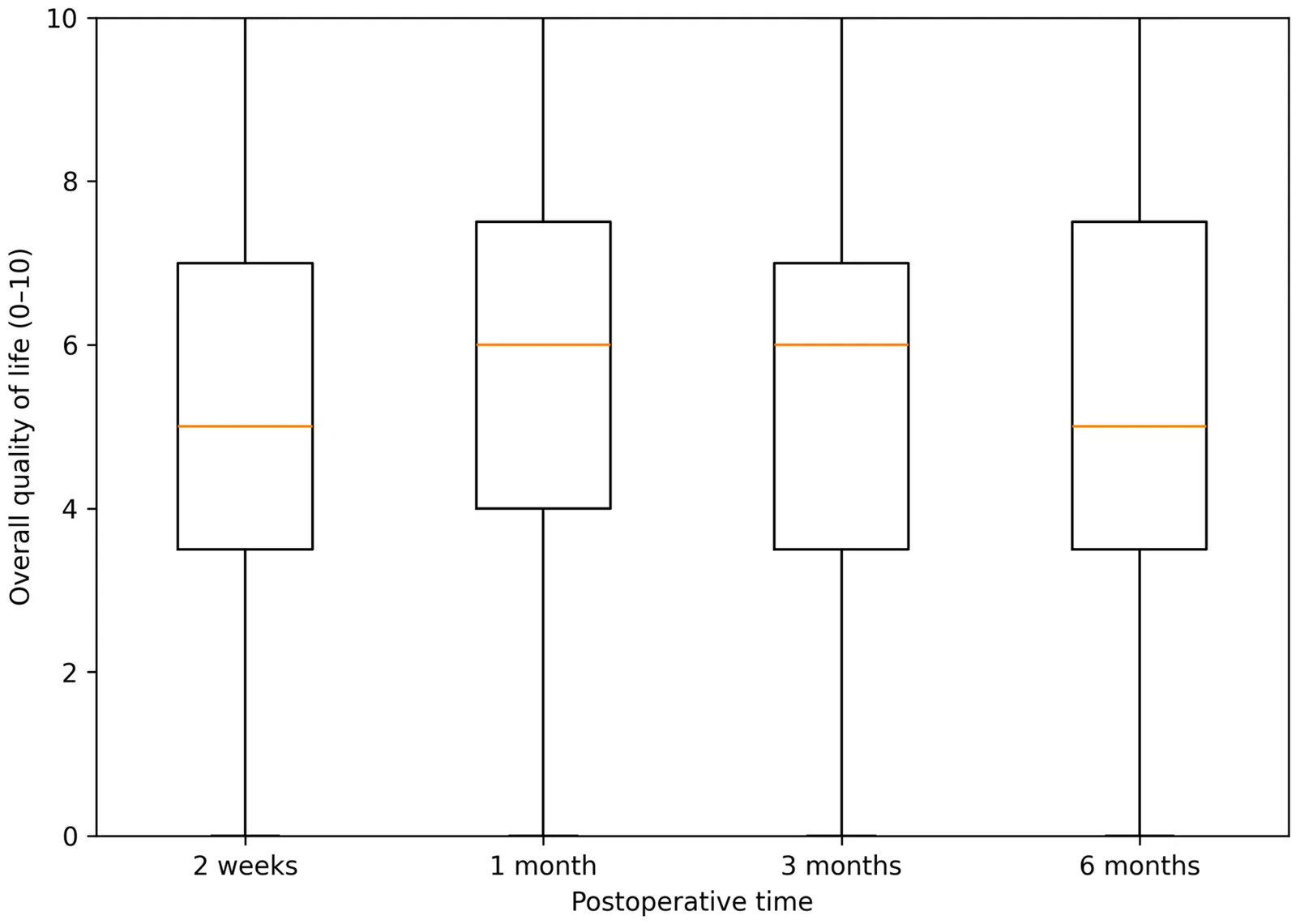
Distribution of patient-reported global quality-of-life scores across four postoperative assessments in 23 patients with temporary intestinal stomas. Overall quality of life (QoL) was assessed using a direct 0–10 rating item at 2 weeks, 1 month, 3 months, and 6 months after stoma formation.

In contrast to the composite index, the direct 0–10 overall quality-of-life item did not show a significant longitudinal change. Median global QoL was 5 at 2 weeks, 6 at 1 month, 6 at 3 months, and 5 at 6 months. The overall difference across timepoints was not statistically significant (Friedman chi-square(3) = 1.20, *p* = 0.753) (Figure 2).

Thus, longitudinal change in the exploratory composite index was not accompanied by a measurable change in patients’ direct rating of overall quality of life. Because the global QoL item also contributed to the composite index, this contrast was further evaluated in robustness analyses.

### 3.4. Exploratory Analysis According to Sex

Male patients had higher patient-level longitudinal composite-index values than female patients. The median patient-level mean score across the four assessments was 14.11 in men and 9.60 in women. The between-sex difference was statistically significant in the exploratory Mann–Whitney analysis (U = 28.0, *p* = 0.024), with a rank-biserial correlation of 0.569.

A sensitivity analysis excluding the two composite components related to sexual activity yielded a similar result. Median patient-level mean scores remained higher in men than in women (13.05 vs. 8.60; U = 30.0, *p* = 0.032; rank-biserial correlation = 0.538), suggesting that the observed sex difference was not solely attributable to the sexual-function items.

Descriptively, the sex difference became more pronounced during follow-up. At 6 months, the median corrected composite index was 16.2 in men compared with 9.6 in women (Supplementary Figure S1). Given the limited sample size and absence of multivariable adjustment, these findings should be interpreted as exploratory.

### 3.5. Association with Age

Age was analysed as a continuous variable. A moderate inverse correlation was observed between age and the patient-level mean composite index, but this association did not reach statistical significance (Spearman rho = −0.344, *p* = 0.108). Accordingly, no statistically significant association between increasing age and the composite outcome could be demonstrated in this cohort.

### 3.6. Stoma Type and Surgical Setting

Analyses according to stoma type and surgical setting were considered descriptive because of the small subgroup sizes. Among patients with ileostomy (*n* = 16), the median composite index was 9.9 at 2 weeks, 10.7 at 1 month, 11.7 at 3 months, and 11.6 at 6 months. Among patients with colostomy (n = 7), corresponding median scores were 8.7, 13.5, 13.0, and 15.8.

Patients undergoing elective surgery (n = 16) had median scores of 9.9, 10.7, 11.7, and 11.6 across the four assessments, whereas those undergoing emergency surgery (n = 7) had median scores of 8.7, 11.7, 13.0, and 14.3, respectively. No formal hypothesis testing was performed for these subgroup comparisons, and the observed differences should be regarded as hypothesis-generating only (Supplementary Table S1).

### 3.7. Functional and Psychosocial Outcomes

Several individual questionnaire items showed descriptive evidence of improved practical adaptation over time (Table 3). The proportion of patients reporting that they were able to manage their stoma outside the home increased from 43.5% (10/23) at 2 weeks to 65.2% (15/23) at 6 months.

Similarly, the proportion reporting a clear limitation of leisure activities decreased from 56.5% (13/23) at 2 weeks to 34.8% (8/23) at both 3 and 6 months. However, substantial psychological and physical limitations persisted. Psychological limitation in the desire to pursue hobbies or sports was reported by 78.3% of patients at both 2 weeks and 1 month and by 69.6% at both 3 and 6 months. Physical limitation in sports or recreational activities decreased from 82.6% at 2 weeks to 69.6% at 6 months.

Interest in peer-support groups remained relatively stable throughout follow-up, ranging from 39.1% to 43.5% of the cohort. These item-level results were considered descriptive and were not subjected to individual inferential testing.

### 3.8. Stoma-Related Complications

Self-reported stoma-related complications were common during follow-up. At least one early complication was reported by 13 of 23 patients (56.5%). The most frequently reported early events were peristomal dermatitis in seven patients (30.4%), surgical-site infection in five (21.7%), stoma detachment in four (17.4%), and either marked oedema or ileus/obstruction in three patients each (13.0%).

At least one late complication was reported by 15 patients (65.2%). Difficulties with appliance management were the most frequent late problem, affecting 10 patients (43.5%), followed by parastomal hernia in five (21.7%) and recurrent dehydration in three (13.0%). Complication data were self-reported and were deduplicated at the patient level across the four assessments. Given the limited cohort size, no formal association between individual complications and the composite outcome was tested (Supplementary Table S2).

### 3.9. Robustness Analyses

Excluding the global QoL component from the composite produced very similar longitudinal results: median values were 8.7, 10.4, 11.8, and 11.5 across the four assessments, respectively, with Friedman chi-square(3) = 19.38 (*p* < 0.001). Bonferroni-adjusted pairwise comparisons remained significant for 2 weeks versus 3 months (*p* = 0.041), 2 weeks versus 6 months (*p* = 0.009), and 1 month versus 6 months (*p* = 0.049).

When the global QoL component and both sexual-activity components were excluded, the overall longitudinal difference also remained significant (Friedman chi-square(3) = 16.74, *p* < 0.001), although only the 2-week versus 6-month comparison remained significant after Bonferroni correction (adjusted *p* = 0.012). Excluding the corrected psychological hobby/sport limitation component entirely did not alter the primary overall result (Friedman chi-square(3) = 19.08, *p* < 0.001). These analyses are summarised in Supplementary Table S4.

## 4. Discussion

### 4.1. Principal Findings

In this prospective longitudinal cohort, the exploratory stoma-related patient-reported composite index changed significantly during the first six postoperative months. Median values were lowest at the earliest assessment, increased at 1 and 3 months, and remained broadly similar at 6 months. Significant adjusted differences were observed between the earliest assessment and later follow-up, whereas no significant difference was observed between 3 and 6 months.

Kendall’s W was 0.276, and individual trajectories varied widely across follow-up. The aggregate pattern is consistent with change in several stoma-related functional and psychosocial dimensions during the early postoperative period, but the non-validated composite does not establish a single latent construct of ‘adaptation’ or quantify validated health-related quality of life. No minimal clinically important difference or other threshold for clinically meaningful change has been established for this index. Statistical significance therefore cannot be equated with clinically meaningful improvement in adaptation or quality of life.

A particularly relevant finding was the different pattern observed for the multidimensional composite and the direct global quality-of-life item. Although the composite index changed significantly, median global QoL remained between 5 and 6 and showed no statistically significant longitudinal change. Importantly, the global QoL item was itself one component of the composite; however, the composite remained significantly different across time after that component was removed, supporting the robustness of the observed multidimensional pattern.

### 4.2. Stoma-Specific Adaptation Versus Global Quality of Life

The structure of the exploratory composite provides a plausible explanation for the divergent trajectories. It incorporated practical stoma management, social functioning, perceived limitations, psychological responses, relationships, autonomy, and other consequences directly related to living with a stoma. These domains overlap conceptually with multidimensional frameworks used by established ostomy-specific QoL instruments [2,3], but the present index has not undergone equivalent psychometric validation.

Accordingly, the increase in the composite should not be described as proof of improvement in validated QoL or as validation of an adaptation construct. A more defensible interpretation is that several stoma-related patient-reported dimensions changed in a favourable direction during follow-up.

The direct global QoL item provides an informative counterpoint. Patients may learn to manage an appliance, leave the home with greater confidence, modify routines, and overcome some practical limitations while still experiencing the consequences of cancer or inflammatory disease, postoperative morbidity, altered body image, sexuality, uncertainty, or other aspects of life that contribute to their overall well-being. Longitudinal and qualitative studies support the concept that individual dimensions of adjustment may evolve differently over time [4,5,6]. This distinction is clinically relevant: improvement in stoma-management competence should not automatically be interpreted as psychological recovery or restoration of global quality of life.

### 4.3. Robustness and Timing of Longitudinal Change

The robustness analyses support an overall longitudinal signal, but the pattern of pairwise differences was less stable when potentially influential components were removed. After exclusion of both the global QoL and sexual-activity components, the overall Friedman test remained significant (chi-square(3) = 16.74, *p* < 0.001), whereas only the 2-week versus 6-month comparison remained significant after Bonferroni correction (adjusted *p* = 0.012). These findings provide greater confidence in the presence of an overall time-related change than in the precise timing or magnitude of differences between individual assessments. Pairwise findings should therefore be interpreted cautiously.

The absence of a statistically significant difference between 3 and 6 months does not establish a plateau or complete adaptation. Persistent psychological and physical limitations and heterogeneous individual trajectories remain relevant, consistent with longitudinal evidence that different domains may change at different rates [4,5]. All assessments were completed with temporary stomas still in situ, so stoma reversal itself cannot explain the observed changes. Reversal is generally considered from approximately 12 weeks in our practice when clinically appropriate, but its timing varies and may be delayed; temporary status does not imply reversal by the 3-month assessment. The expectation of future reversal may nevertheless influence patients’ psychological experience.

### 4.4. Functional and Psychosocial Outcomes

Several individual outcomes provide further context for the longitudinal composite index. The proportion of patients able to manage their stoma outside the home increased from 43.5% at 2 weeks to 65.2% at 6 months, while the proportion reporting a clear limitation of leisure activities decreased from 56.5% to 34.8%. These findings suggest increasing functional independence. Nevertheless, the residual burden remained substantial: at 6 months, approximately 70% of patients continued to report psychological limitation in pursuing hobbies or sports, and a similar proportion reported physical limitation of recreational activities.

Large observational studies have likewise shown that stoma-related problems extend beyond appliance management. Difficulties with social and physical activities, leakage, body profile, and access to specialised stoma care are common concerns among people living with a stoma [7]. The results therefore support a clinical distinction between technical self-management and broader psychosocial reintegration. A patient who can independently manage a stoma may nevertheless remain significantly restricted in social, recreational, physical, or intimate aspects of life.

An important consideration in interpreting our findings is the clinical heterogeneity of the study population. Patients undergoing stoma formation for malignant disease may experience psychological and functional burdens related not only to the stoma itself but also to their underlying diagnosis, oncological treatments, and concerns regarding prognosis. Conversely, patients with benign conditions may experience different patterns of physical and psychological adaptation. Consequently, the observed longitudinal changes in patient-reported outcomes should not be interpreted as exclusively attributable to stoma-related adaptation, as disease-specific and treatment-related factors may have contributed to these trajectories.

### 4.5. Exploratory Subgroup Observations

Exploratory analyses suggested higher longitudinal composite-index values in men than in women, whereas increasing age showed a moderate but non-significant inverse association with the composite. Descriptive differences were also observed according to stoma type and surgical setting. The sex comparison included only 13 women and 10 men, and the cohort was too small to permit meaningful multivariable adjustment for age, underlying disease, stoma type, surgical setting, complications, or other potential confounders. Ileostomy versus colostomy and elective versus emergency surgery comparisons were descriptive only. These findings are hypothesis-generating and do not establish independent prognostic effects.

Prior research has described sex-related concerns, body-image and sexual outcomes, and heterogeneous age-related findings [8,9,10,11,12]. Studies of emergency and planned stoma surgery also highlight the relevance of clinical context, expectations, and preparation [13,14]. These observations provide context but cannot resolve confounding in the present cohort; larger prospective studies are needed.

### 4.6. Clinical Context and Postoperative Support

Self-reported stoma-related problems were common during follow-up, particularly peristomal dermatitis and appliance-management difficulties. Because these events were not independently adjudicated, they represent patient-reported complication burden rather than formal incidence estimates. Previous studies have also described skin complications, leakage, and associated restrictions in daily activities [7,15,16]. Together with persistent psychological and activity-related limitations despite increasing practical independence, these findings suggest that postoperative assessment should extend beyond technical stoma management.

Approximately 40% of participants expressed interest in peer support, which indicates perceived need rather than intervention effectiveness. Qualitative research emphasises information and support [14], and reviews of remote educational interventions report benefits for selected outcomes with variable effects on global quality of life [17,18]. The present study was not designed or powered to evaluate supportive interventions or determine whether individual complications independently predicted the composite outcome; these observations remain descriptive.

### 4.7. Strengths and Limitations

The principal strength of this study is its prospective longitudinal design, with repeated assessment of the same patients at four predefined timepoints during the first six postoperative months. Complete observations were available for all 23 patients included in the final analysis, permitting within-patient evaluation of longitudinal change. The study also incorporated practical, social, psychological, and stoma-specific experiences rather than relying solely on one global rating. Analysis of the global QoL item separately from the composite allowed an informative comparison between multidimensional stoma-related outcomes and directly reported overall quality of life.

Several limitations must be acknowledged. First, the cohort was small and derived from a single centre, limiting precision, subgroup analyses, and generalisability. The analytical file contained the 23 complete cases used for the manuscript but did not permit reliable reconstruction of the number initially approached or enrolled and subsequently lost or excluded; participant flow before derivation of the complete-case cohort could therefore not be fully described, and attrition or selection bias cannot be excluded. Repeated within-patient measurements do not overcome the limitations of the small sample, and no formal a priori sample-size calculation was performed.

Second, the modified questionnaire and composite index were not formally psychometrically validated. This is the principal measurement limitation of the study. Established ostomy-specific instruments have undergone formal validation [2,3], whereas the present index should be regarded only as an exploratory composite of heterogeneous stoma-related patient-reported items. Equal weighting of components assumes, without empirical validation, that each contributes similarly to the composite. No minimal clinically important difference has been established, precluding translation of statistically significant changes into a quantified clinical benefit.

Third, the direct global QoL item contributed to the composite while also being analysed separately, so the measures were not independent. The persistence of the overall longitudinal result after removing this component reduces, but does not eliminate, concerns about construct overlap. The inherited sexual-satisfaction coding assigned 0 to other/non-applicable responses, although non-applicability does not necessarily indicate an unfavourable outcome. Retaining this rule preserved the original scoring framework but remains a measurement limitation. Excluding both sexual-activity components together with global QoL preserved the significant overall time effect, while only the 2-week versus 6-month comparison remained significant after correction; the pairwise pattern is therefore sensitive to component selection.

Fourth, the study relied on patient-reported data and was susceptible to recall, social-desirability, and response-interpretation biases. In addition, responses were manually transcribed into the electronic database after the original follow-up assessments, creating a potential risk of transcription error. Database timestamps therefore represent data-entry dates rather than assessment dates; the reanalysis relied on the predefined postoperative assessment labels and an audited patient-level dataset.

Fifth, no preoperative patient-reported baseline was available. The study therefore describes change between postoperative assessments and cannot estimate the pre- to post-stoma effect of stoma formation or distinguish stoma-specific change from general postoperative recovery and changes related to the underlying disease. The population was also clinically heterogeneous, including ileostomies and colostomies, elective and emergency procedures, and several underlying diagnoses. Patients with malignant disease may experience additional psychological distress and treatment-related burdens associated with cancer diagnosis, adjuvant therapies, and prognostic uncertainty, whereas patients with benign conditions may follow substantially different clinical and psychological trajectories. These differences represent potential confounding factors that could not be adequately accounted for in the present analysis.

Sixth, all 23 complete cases had temporary stomas, and all four assessments were completed with the stoma in situ. This excludes post-reversal status as an explanation for the observed longitudinal changes but limits generalisability to patients with permanent stomas or after reversal. The expectation of future reversal may itself influence patient-reported experiences. Clinical heterogeneity and the small sample prevented meaningful adjusted analyses; sex and age findings were exploratory and unadjusted and do not establish independent prognostic effects. Finally, complication data were self-reported and not independently adjudicated according to standard clinical definitions, and represent patient-reported burden rather than formal incidence.

## 5. Conclusions

In this complete-case cohort of 23 patients with temporary stomas, the exploratory stoma-related patient-reported composite showed statistically significant longitudinal change during the first six postoperative months, while direct global quality-of-life ratings did not show a parallel change. All assessments were completed with the stoma in situ. The overall longitudinal signal persisted in robustness analyses, but the pattern of pairwise differences was less stable after exclusion of selected components.

These findings suggest that individual stoma-related dimensions may evolve differently from global quality of life. They do not establish clinically meaningful improvement in adaptation or quality of life, because the composite was not validated and no clinically meaningful change threshold is available. The small, single-centre, clinically heterogeneous cohort and absence of a preoperative baseline further limit interpretation. Larger prospective studies using validated patient-reported outcome measures, with prospective sample-size determination and explicit assessment of temporary and permanent stomas before and after reversal, are needed to confirm these exploratory findings.

## Figures and Tables

**Table 1:** Baseline demographic and clinical characteristics of the 23 patients with temporary intestinal stomas included in the complete-case longitudinal analysis.

Characteristic	Overall (N = 23)
Age, years	63 (52–71)
Age range, years	26–87
BMI	23.4 (20.2–25.6)
Sex	
Female	13 (56.5%)
Male	10 (43.5%)
Stoma type	
Ileostomy	16 (69.6%)
Colostomy	7 (30.4%)
Stoma permanence	
Temporary	23 (100%)
Permanent	0 (0%)
Surgical setting	
Elective	16 (69.6%)
Emergency	7 (30.4%)
Indication for stoma	
Oncological disease	16 (69.6%)
Inflammatory bowel disease	4 (17.4%)
Diverticular disease	3 (13.0%)

Data are presented as median (IQR) or n (%), unless otherwise stated. BMI, body mass index.

**Table 2:** Longitudinal changes in the corrected exploratory stoma-related patient-reported composite index among 23 patients with temporary intestinal stomas at 2 weeks, 1 month, 3 months, and 6 months after stoma formation.

Timepoint	N	Median	Q1	Q3	IQR	Min	Max
2 weeks	23	9.4	7.2	13.6	6.4	1.5	27.0
1 month	23	10.9	8.7	13.6	4.9	2.0	27.5
3 months	23	12.2	9.3	15.3	6.0	4.0	27.7
6 months	23	11.8	9.3	17.2	7.9	3.0	26.5
Pairwise Comparison		Wilcoxon Statistic	Raw *p*	Bonferroni-Adjusted *p*		Median Within-Patient Change	
2 weeks vs. 1 month		64.0	0.073	0.441		0.5	
2 weeks vs. 3 months		48.0	0.006	0.037		1.4	
2 weeks vs. 6 months		39.5	0.003	0.016		2.0	
1 month vs. 3 months		54.0	0.019	0.111		1.2	
1 month vs. 6 months		50.0	0.007	0.045		1.1	
3 months vs. 6 months		46.0	0.255	1.000		0.0	

Global longitudinal test: Friedman chi-square(3) = 19.08, *p* < 0.001; Kendall’s W = 0.276. Higher composite-index values indicate more favourable responses according to the study scoring algorithm. Pairwise interpretation is based on Bonferroni-adjusted *p*-values across six manuscript comparisons.

**Table 3:** Longitudinal changes in selected functional and psychosocial patient-reported outcomes among 23 patients with temporary intestinal stomas across four postoperative assessments.

Outcome	2 Weeks n (%)	1 Month n (%)	3 Months n (%)	6 Months n (%)
Able to manage stoma outside home	10 (43.5%)	9 (39.1%)	14 (60.9%)	15 (65.2%)
Clear leisure-activity limitation	13 (56.5%)	11 (47.8%)	8 (34.8%)	8 (34.8%)
Psychological limitation of hobbies/sports	18 (78.3%)	18 (78.3%)	16 (69.6%)	16 (69.6%)
Physical limitation of sports/recreation	19 (82.6%)	19 (82.6%)	17 (73.9%)	16 (69.6%)
Would find a peer-support group useful	10 (43.5%)	9 (39.1%)	9 (39.1%)	10 (43.5%)

Values are descriptive only; no item-level inferential tests were performed. Partial leisure limitation is not included in the clear-limitation row.

## Data Availability

The data presented in this study are available on request from the authors.

## Supplementary Materials

The following supporting information can be downloaded at: https://www.mdpi.com/article/10.3390/jcm15218654/s1, Table S1: Longitudinal distribution of the corrected exploratory stoma-related patient-reported composite index according to stoma type and surgical setting in the 23-patient analytic cohort; Table S2: Frequency and distribution of self-reported stoma-related complications during postoperative follow-up among 23 patients with temporary intestinal stomas; Figure S1: Sex-stratified longitudinal trajectories of the corrected exploratory stoma-related patient-reported composite index across four postoperative assessments in patients with temporary intestinal stomas; Table S3: Components and scoring rules for the exploratory composite index; Table S4: Post hoc robustness analyses of the longitudinal composite index.
