# Supplementary material for: Patient-Reported Trajectories After Intestinal Stoma Formation: A Prospective Longitudinal Study of Stoma-Related Outcomes and Global Quality of Life
Source: J Clin Med. 2026 Nov 6;15(21):8654. doi: 10.3390/jcm15218654 (PMC13764121; doi:10.3390/jcm15218654)
Supplement: Supplementary file 1 [file jcm-15-08654-s001.zip › jcm-4543047-supplementary.pdf]

Supplementary material

Supplementary Table S1. Longitudinal distribution of the corrected exploratory stoma-related patient-reported composite index according to stoma type and surgical setting in the 23-patient analytic cohort.

Group	n	2 weeks median (IQR)	1 month median (IQR)	3 months median (IQR)	6 months median (IQR)
Ileostomy	16	9.9 (6.9-13.5)	10.7 (8.2-12.2)	11.7 (8.9-14.8)	11.6 (8.9-14.9)
Colostomy	7	8.7 (8.5-12.2)	13.5 (10.1-15.1)	13.0 (10.9-15.3)	15.8 (11.8-17.9)
Elective surgery	16	9.9 (6.9-14.8)	10.7 (8.2-14.9)	11.7 (8.9-16.7)	11.6 (8.9-17.0)
Emergency surgery	7	8.7 (8.6-12.2)	11.7 (9.6-13.6)	13.0 (11.9-14.6)	14.3 (12.3-16.8)

These subgroup analyses are descriptive and hypothesis-generating only; no formal between-group hypothesis tests were performed because of small subgroup sizes.

Supplementary Table S2. Frequency and distribution of self-reported stoma-related complications during postoperative follow-up among 23 patients with temporary intestinal stomas.

Complication	Patients, n (%)	Type
Any early complication	13 (56.5%)	Early
Peristomal dermatitis	7 (30.4%)	Early
Surgical-site infection	5 (21.7%)	Early
Stoma detachment	4 (17.4%)	Early
Marked oedema	3 (13.0%)	Early
Ileus/obstruction	3 (13.0%)	Early
Dehydration/electrolyte imbalance	2 (8.7%)	Early
Any late complication	15 (65.2%)	Late
Appliance-management problems	10 (43.5%)	Late
Parastomal hernia	5 (21.7%)	Late
Recurrent dehydration	3 (13.0%)	Late
Stomal stenosis	1 (4.3%)	Late
Late retraction	1 (4.3%)	Late
Peristomal granulomas/ulceration	1 (4.3%)	Late

Complications were self-reported and deduplicated at the patient level across follow-up. Categories with one patient are reported for completeness.

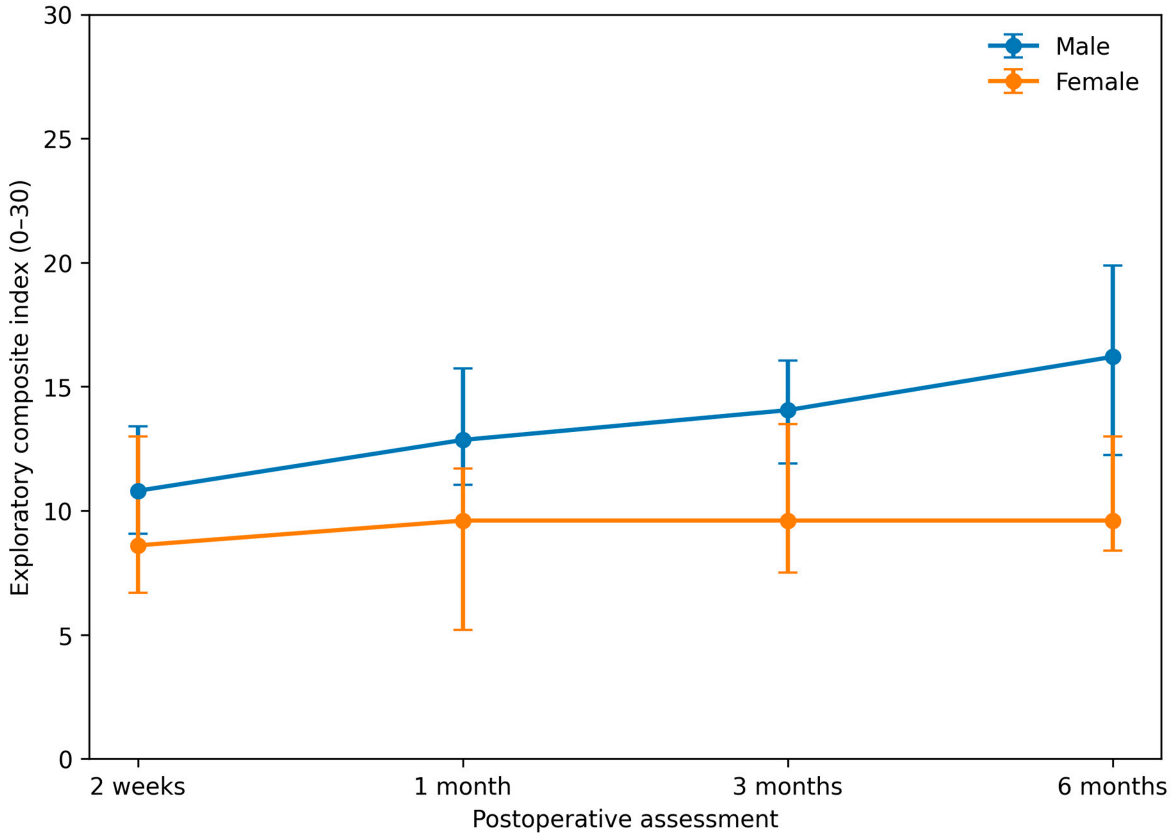

Supplementary Figure S1. Sex-stratified longitudinal trajectories of the corrected exploratory stoma-related patient-reported composite index across four postoperative assessments in patients with temporary intestinal stomas.

Supplementary Table S3. Components and scoring rules for the exploratory composite index

Component	Questionnaire domain/item	Scoring rule
1	Depression after surgery	No=1; Yes=0
2	Pre-/postoperative sexual-activity status	1 when preoperative and postoperative activity status were the same; 0 when different
3	Sexual satisfaction	Yes=1; other/non-applicable response=0
4	Clothing problems due to stoma	No=1; Yes=0
5	Diet adaptation due to stoma	No=1; Yes=0
6	Diet modification to avoid gas in public	No=1; Yes=0
7	Daily stoma-care time	<3 min=1; 3-5 min=0.5; >5 min=0
8	Problems managing the stoma	No=1; any affirmative response=0
9	Ability to manage stoma outside home	Yes=1; No=0
10	Leisure limitation	No limitation=1; partial limitation=0.5; clear limitation=0
11	Psychological limitation of hobbies/sports	No=1; any affirmative response=0
12	Physical limitation of sport/recreation	No=1; Yes=0
13	Difficulty adapting to the stoma	1 - raw score/10
14	Embarrassment caused by the stoma	1 - raw score/10
15	Overall quality of life	raw score/10
16	Difficulty looking at the stoma	1 - raw score/10
17	Difficulty caring for the stoma	1 - raw score/10
18	Sense of control over life	raw score/10
19	Depression due to the stoma	1 - raw score/10
20	Difficulty meeting new people	1 - raw score/10
21	Financial burden	1 - raw score/10
22	Family stress	1 - raw score/10
23	Interference with travel	1 - raw score/10
24	Interference with personal relationships	1 - raw score/10
25	Isolation	1 - raw score/10
26	Sufficiency of support from family/friends	raw score/10
27	Interference with social activities	1 - raw score/10
28	Interference with intimacy	1 - raw score/10

Component	Questionnaire domain/item	Scoring rule
29	Uncertainty about the future	1 - raw score/10
30	Acceptance of the stoma	raw score/10

All 30 components were equally weighted and summed. The index range is 0-30. The table documents the scoring algorithm used for the manuscript reanalysis; it does not imply psychometric validation of the composite construct. Sexual-satisfaction coding (Yes=1; other/non-applicable=0) was inherited from the original spreadsheet. Non-applicability does not necessarily indicate an unfavourable outcome, and this coding is a limitation of the exploratory composite. The primary coding was retained; a post hoc robustness analysis excluded both sexual-activity components together with global QoL (Supplementary Table S4).

Supplementary Table S4. Post hoc robustness analyses of the longitudinal composite index

Analysis	Median values: 2 wk ǀ 1 mo ǀ 3 mo ǀ 6 mo	Friedman chi-square	p-value	Key Bonferroni-adjusted pairwise findings
Primary corrected 30-component index	9.4 ǀ 10.9 ǀ 12.2 ǀ 11.8	19.08	<0.001	2 wk vs 3 mo p=0.037; 2 wk vs 6 mo p=0.016; 1 mo vs 6 mo p=0.045
Excluding global QoL component	8.7 ǀ 10.4 ǀ 11.8 ǀ 11.5	19.38	<0.001	2 wk vs 3 mo p=0.041; 2 wk vs 6 mo p=0.009; 1 mo vs 6 mo p=0.049
Excluding global QoL + sexual-activity components	8.7 ǀ 8.9 ǀ 11.4 ǀ 11.4	16.74	<0.001	2 wk vs 6 mo p=0.012; other comparisons not significant after correction
Excluding psychological hobby/sport limitation component	9.4 ǀ 10.7 ǀ 12.2 ǀ 11.8	19.08	<0.001	Overall result unchanged; pairwise tests not used for primary interpretation

Robustness analyses were performed after audit of the original dataset and scoring logic and are therefore post hoc. They are presented to evaluate dependence of the main longitudinal result on specific composite components.
